# Mechanisms of radiation‐induced tissue damage and response

**DOI:** 10.1002/mco2.725

**Published:** 2024-09-20

**Authors:** Lin Zhou, Jiaojiao Zhu, Yuhao Liu, Ping‐Kun Zhou, Yongqing Gu

**Affiliations:** ^1^ Beijing Key Laboratory for Radiobiology Beijing Institute of Radiation Medicine Beijing China; ^2^ Hengyang Medical College University of South China Hengyang Hunan China; ^3^ College of Life Sciences Hebei University Baoding China

**Keywords:** mechanisms, radiation‐induced tissue injury, single‐cell sequencing, therapeutic target

## Abstract

Radiation‐induced tissue injury (RITI) is the most common complication in clinical tumor radiotherapy. Due to the heterogeneity in the response of different tissues to radiation (IR), radiotherapy will cause different types and degrees of RITI, which greatly limits the clinical application of radiotherapy. Efforts are continuously ongoing to elucidate the molecular mechanism of RITI and develop corresponding prevention and treatment drugs for RITI. Single‐cell sequencing (Sc‐seq) has emerged as a powerful tool in uncovering the molecular mechanisms of RITI and for identifying potential prevention targets by enhancing our understanding of the complex intercellular relationships, facilitating the identification of novel cell phenotypes, and allowing for the assessment of cell heterogeneity and spatiotemporal developmental trajectories. Based on a comprehensive review of the molecular mechanisms of RITI, we analyzed the molecular mechanisms and regulatory networks of different types of RITI in combination with Sc‐seq and summarized the targeted intervention pathways and therapeutic drugs for RITI. Deciphering the diverse mechanisms underlying RITI can shed light on its pathogenesis and unveil new therapeutic avenues to potentially facilitate the repair or regeneration of currently irreversible RITI. Furthermore, we discuss how personalized therapeutic strategies based on Sc‐seq offer clinical promise in mitigating RITI.

## Graphical abstract

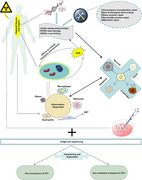

## 1 INTRODUCTION

Radiotherapy, an essential clinical treatment for many malignant tumors, involves cancer patients undergoing repeated radiotherapy over an extended period.^1^ While targeting the diseased area, radiotherapy may impact the normal tissue surrounding the tumor, leading to radiation (IR)‐induced tissue injury (RITI).^2^, ^3^, ^4^, ^5^ This complication has shown a significant increase in incidence alongside the widespread use of radiotherapy in clinical settings. RITI encompasses a variety of pathological processes, including inflammation regulatory,^6^, ^7^, ^8^ oxidative stress,^9^, ^10^, ^11^ cell damage (apoptosis,^12^, ^13^, ^14^ senescence,^15^, ^16^, ^17^ pyroptosis,^18^, ^19^, ^20^ and ferroptosis^21^, ^22^, ^23^), as well as extracellular matrix (ECM) remodeling and fibrosis.^24^, ^25^, ^26^ Considerable research efforts have focused on understanding the mechanisms and therapeutic strategies in RITI. The prevention and therapy of RITI present significant challenges due to the intricate complexity of RITI and the interconnections among various pathological signaling pathways. Therefore, to identify the mechanism of RITI and therapy target is essential for the effective application of radiotherapy. RITI is usually the result of DNA damage,^27^ oxidative stress signals^28^, ^29^, ^30^ and remodeling of tissue microenvironment by synergistic activation of various inflammatory response signals,^2^, ^31^ cell injury,^21^, ^32^, ^33^, ^34^, ^35^, ^36^ and damage repair signals by key target cell injury and major effector cells.^37^, ^38^, ^39^ Although many studies have attempted to elucidate the pathogenesis of RITI and explore potential intervention targets, the specific prevention and treatment of RITI is still a great challenge due to this multicellular and multi‐factor mediated effect.

Single‐cell sequencing (Sc‐seq) is a new technology that has rapidly developed in the past decade, allowing for the comprehensive analysis of the genome, transcriptome, and epigenetic inheritance of individual cells.^40^ With the development of the third‐generation high‐throughput sequencing technology, coupled with the declining sequencing costs, have propelled the widespread adoption of Sc‐seq.^41^ The integration of Sc‐seq has brought about transformative changes within the field of omics by enabling the in‐depth exploration of cell populations.^42^, ^43^ This technology has a unique capacity to discern the heterogeneity within cell populations, identify previously unknown cellular sub‐populations, and elucidate the peculiarity of individual cells,^44^ which has greatly expanded researchers’ understanding of cell typing and functional research.^45^, ^46^, ^47^ Recently, Sc‐seq has been widely used in developmental and pathological research, such as development,^48^, ^49^ neurobiology,^50^ immunology,^51^, ^52^, ^53^, ^54^ microbiology,^55^ and cancer.^56^, ^57^ Particularly in the case of rare cells, Sc‐seq emerges as a powerful tool for achieving heightened precision and deeper exploration, thereby underscoring its pivotal role in advancing cellular research.^58^, ^59^ Consequently, based on the ability of Sc‐seq to identify gene characterization or cell subtypes at the single‐cell level, it may be a powerful strategy for identifying potential intervention targets for RITI.

Currently, significant challenges remain in the prevention and treatment of RITI. The application of Sc‐seq to RITI is of great significance in elucidating the key mechanism of RITI and seeking specific therapeutic targets. This paper reviews the molecular mechanisms underlying RITI and explores the latest advancements in the application of Sc‐seq to this field. We aim to identify potential mechanisms and candidate therapeutic targets for RITI. Additionally, we discuss the limitations of Sc‐seq and how current studies are addressing these challenges. Ultimately, our goal is to enhance the understanding of RITI and contribute to the search for more effective management strategies for radioactive tissue damage.

## 2 MECHANISM OF RITI

### 2.1 DNA damage and repair mechanisms

Exposure to IR can cause direct or indirect damage to cellular DNA, leading to various forms of injury, including DNA double strand breaks (DSB), base damage, and cross‐linking.^60^ In response to this damage, cells activate DNA repair mechanisms, such as nucleotide excision repair (NER) and DSB repair, to restore DNA integrity.^60^, ^61^ However, if the extent of IR damage surpasses the cell's repair capacity, it can result in severe consequences, including cell death, mutations, and carcinogenesis.^62^, ^63^ Additionally, DNA damage may initiate apoptosis, or programmed cell death (PCD), as a protective measure against the uncontrolled proliferation of damaged cells that could develop into tumors.^64^ Thus, DNA damage is a critical factor in RITI. The ability of cells to repair this damage directly influences their survival and functionality, highlighting the importance of mitigating the adverse effects of IR exposure.

#### 2.1.1 Types of DNA damage

##### DNA double‐strand breaks

DSB is the most severe form of DNA damage and can directly trigger PCD, such as apoptosis.^65^ If these breaks, particularly those induced by IR, are not repaired promptly, they can disrupt the accurate transmission of genetic information. This disruption may lead to gene mutations, cell death, and various diseases, including cancer.^66^, ^67^, ^68^ Furthermore, unaddressed DSB can result in chromosome breakage, leading to chromosomal abnormalities or the loss of genetic information.^69^ Additionally, such damage may induce cell cycle arrest, thereby affecting cell growth and division, or directly cause cellular senescence.^70^, ^71^, ^72^ In summary, the outcome of cellular fate following a DSB is a complex process influenced by the cell's self‐repair capabilities, the external environment, and various regulatory mechanisms. Research has confirmed that DSB induced by IR significantly contribute to injuries in skin, brain, and lung tissues. Therefore, it is crucial to target these breaks and preserve DNA integrity in the context of IR exposure.

##### DNA base damage

DNA base damage (DBD) refers to alterations or damage to the bases within the DNA molecule. This damage can arise from various sources, including internal and external chemicals, IR, environmental factors, and metabolites produced by the cell itself.^73^, ^74^ Common forms of DBD include base oxidation, alkylation of deoxyribonucleotides, single base pair breaks, and base deletions or mismatches.^75^, ^76^ Such damage is critical in the development of various diseases, as it can compromise the integrity of genetic information and disrupt normal gene function.^77^ Consequently, this increases the risk of mutations, which can lead to cancer and other health issues.

Cells possess a sophisticated DNA repair system designed to rectify base damage. However, if the damage is extensive or if the repair mechanisms fail, errors may occur during DNA repair and replication, resulting in mutations.^77^ Furthermore, DBD is linked to the onset of numerous diseases, including neurodegenerative disorders, and diabetes.^78^, ^79^ This damage can also initiate apoptosis, inflammatory responses, and tissue damage, contributing to the progression of these conditions.^70^, ^80^ The implications of DBD in the context of RITI have garnered significant attention. Therefore, understanding DBD and its associated repair mechanisms is crucial for developing strategies to prevent and treat RITI.

##### DNA cross‐linking

DNA damage induced by IR encompasses DNA cross‐linking (DCL), which primarily occurs in two forms: DNA–DNA cross‐linking and DNA–protein cross‐linking.^76^, ^81^ In DNA–DNA cross‐linking, bases on the same DNA strand or between two DNA strands can become covalently bonded. In DNA–protein cross‐linking, DNA can be covalently linked to various proteins, including histones, non‐histone proteins in chromatin, regulatory proteins, and enzymes involved in replication and transcription.^82^ This cross‐linking serves as the molecular basis for the chromosomal aberrations observed under a microscope following exposure to IR. The immediate consequence of cross‐linking damage is the disruption of the ongoing replication fork, which impairs normal DNA replication in cells. Furthermore, DCL can lead to mutations and promote cancerous transformations, thereby increasing cancer risk.^83^, ^84^ In the process of RITI, DCL not only directly induces cell death but also activates the DNA repair system. This activation triggers a cascade of cell signaling and apoptosis pathways, further compromising cell survival and function while exacerbating the tissue damage response.^85^

#### 2.1.2 Cellular repair pathways

To maintain the stability and functional integrity of their genomes under various stress conditions, cells have developed complex repair mechanisms to counteract DNA damage. These mechanisms include homologous recombination repair (HR), non‐homologous end‐joining (NHEJ), base excision repair (BER), nucleotide excision repair (NER), and mismatch repair (MMR). Each of these pathways plays a crucial role in ensuring genomic stability.

##### Homologous recombination repair

**FIGURE 1:**
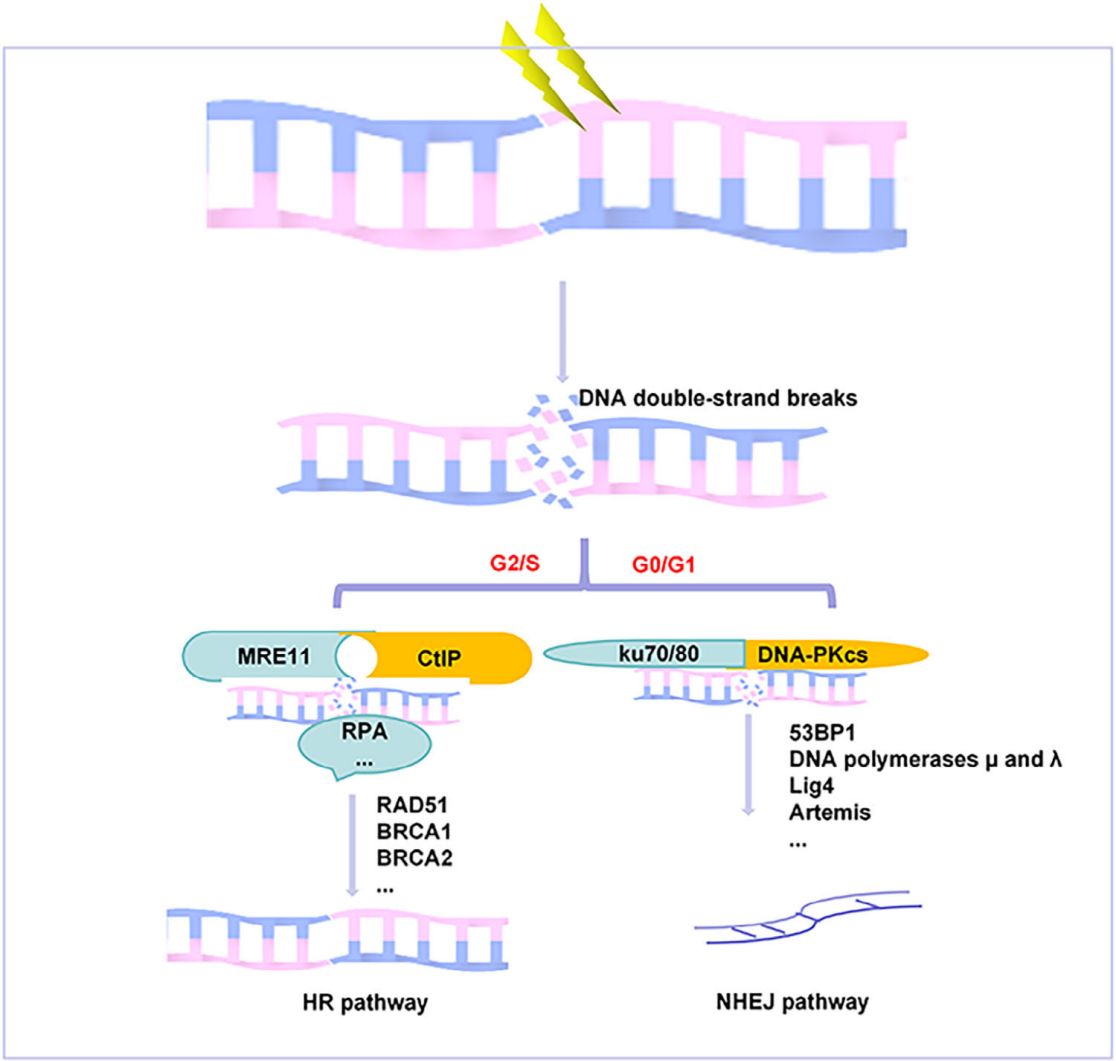
The main mechanism of DNA double‐strand break repair. The homologous recombination (HR) pathway accurately active in the later S and G2 phases and exhibits a lower error rate, as it also utilizes homologous sequences as templates. The nonhomologous end‐joining (NHEJ) pathway is characterized by its speed and propensity for errors, predominantly occurring during the G0/G1 phase.

HR is the primary repair pathway for DSB in DNA (Figure 1). This process relies on homologous sequences, such as homologous chromosomes, sister chromatids, and repetitive DNA sequences, as templates for repair. The preferred template is the homologous sequences of sister chromatids, which is why HR repair predominantly occurs during the S and G2 phases of the cell cycle.^86^, ^87^ Compared with NHEJ, HR repair is more accurate and exhibits a lower error rate, as it also utilizes homologous sequences as templates.^63^ When a DSB occurs, the MRN complex recognizes the damaged site and recruits additional proteins. Nucleases, including MRE11 and CtIP, prune the ends of the DNA to generate single‐stranded DNA.^88^, ^89^ This single‐stranded DNA is quickly bound by replication factor A (RPA), a single‐stranded DNA binding protein, which prevents degradation and the formation of secondary structures.^90^ Subsequently, the RPA‐bound single‐stranded DNA is replaced by the recombination protein RAD51, facilitated by BRCA2. This exchange results in the formation of a stable ssDNA–RAD51 nucleoprotein filament.^91^ Once the RAD51 filament is established, homologous sequence matching occurs. Upon successful matching, the ssDNA–RAD51 complex binds to and invades the site of the DSB, initiating the opening of the break and forming a D‐loop structure.^92^ Ultimately, the homologous sequence is utilized to complete the DNA damage repair process.

##### Non‐homologous end‐joining

NHEJ is a primary mechanism for repairing DNA DSB.^87^ This repair mode is characterized by its speed and propensity for errors, predominantly occurring during the G0/G1 phase of the cell cycle^93^ (Figure 1). Upon detection of a DSB, the Ku70/Ku80 heterodimer swiftly binds to the damaged DNA site, providing protection and stabilization to the DNA ends.^94^, ^95^ Subsequently, two DNA‐dependent protein kinase catalytic subunits (DNA–PKcs) are recruited by the Ku70/Ku80 complex, forming the DNA–PK complex. This complex prevents end clipping and facilitates the inward translocation of the Ku heterodimer along the DNA strand, allowing DNA–PKcs to access the DSB terminus directly. This access is crucial for activating the catalytic activity of DNA–PKcs.^96^, ^97^ However, not all broken DNA ends are amenable to NHEJ repair. Therefore, after the recruitment of DNA–PKcs and Ku70/Ku80, additional nucleases, such as Artemis, CtIP, and EXO1, are necessary for processing the DNA ends. The Artemis‐DNA–PKcs complex serves as the primary nuclease in this context.^98^ Following terminal clipping, DNA polymerases μ and λ (Polμ and Polλ) bind to the DNA to facilitate synthesis, and DNA ligase IV (Lig4) is subsequently employed to finalize the NHEJ repair process.^99^

##### Base excision repair

BER primarily occurs during the G1 phase of the cell cycle.^100^ This mechanism addresses minor damage or errors in the DNA strand, specifically targeting alkylated or oxidized bases, such as xanthine and 7‐methylguanosine.^101^, ^102^ The process begins when DNA glycosylase recognizes and excises the damaged base, creating an apurinic/apyrimidinic (AP) site. Subsequently, AP endonuclease cleaves the phosphodiester bond at the AP site, resulting in a single‐strand break in the DNA.^103^ Following this, DNA polymerase is recruited to the damaged site to synthesize the missing base. Finally, DNA ligase facilitates the connection of the adjacent base, thereby completing the BER process.^104^

##### Nucleotide excision repair

NER is the primary mechanism for removing extensive DNA damage in mammals.^105^ This process utilizes adjacent non‐damaged DNA as a template for repair, which ensures efficiency, accuracy, controllability, universality, low toxicity, and reversibility. NER is categorized into two subpathways: global genomic NER (GG‐NER) and transcription‐coupled NER (TC‐NER).^106^ GG‐NER operates throughout the genome, independent of transcription, and is capable of repairing damage in both transcribed and non‐transcribed DNA strands across active and inactive genes.^107^ In this pathway, several “damage sensing” proteins, including DNA binding and the XPC–Rad23B complex, continuously scan the genome for helical distortions to initiate the repair process. In contrast, TC‐NER specifically addresses damage within the transcription machinery of active genes.^108^ This pathway is initiated when RNA polymerase II binds to the damage site. Subsequently, the proteins CSA, CSB, and XAB2 are recruited to the site. The CSA and CSB complex alter the conformation of RNA polymerase II, facilitating the recruitment of TFIIH. Following this recruitment, the repair process proceeds similarly to that of GG‐NER.

##### Mismatch repair

MMR is a highly conserved biological pathway essential for maintaining genomic stability. This mechanism begins by separating the template strand from the newly synthesized strand.^109^ The MutS protein dimer then binds to the mismatched base on the newly synthesized DNA.^109^ Following this, the MutL2 and MutH proteins collaborate to create a specific incision. The MutL2/MutS2/MutH complex subsequently translocates from the incision site to the mismatch. During this process, the UvrD helicase unwinds the double helix structure of DNA.^110^ Concurrently, an exonuclease hydrolyzes the newly synthesized DNA, including the mismatched bases, until it reaches the point where the mismatched bases are located. Finally, DNA polymerase and DNA ligase resynthesize the excised DNA sequence. DNA MMR is involved in various biological processes, including the maintenance of genome stability, cell apoptosis, and mitotic recombination.^111^, ^112^, ^113^

### 2.2 Oxidative stress and reactive oxygen species

#### 2.2.1 Generation of reactive oxygen species by IR

**FIGURE 2:**
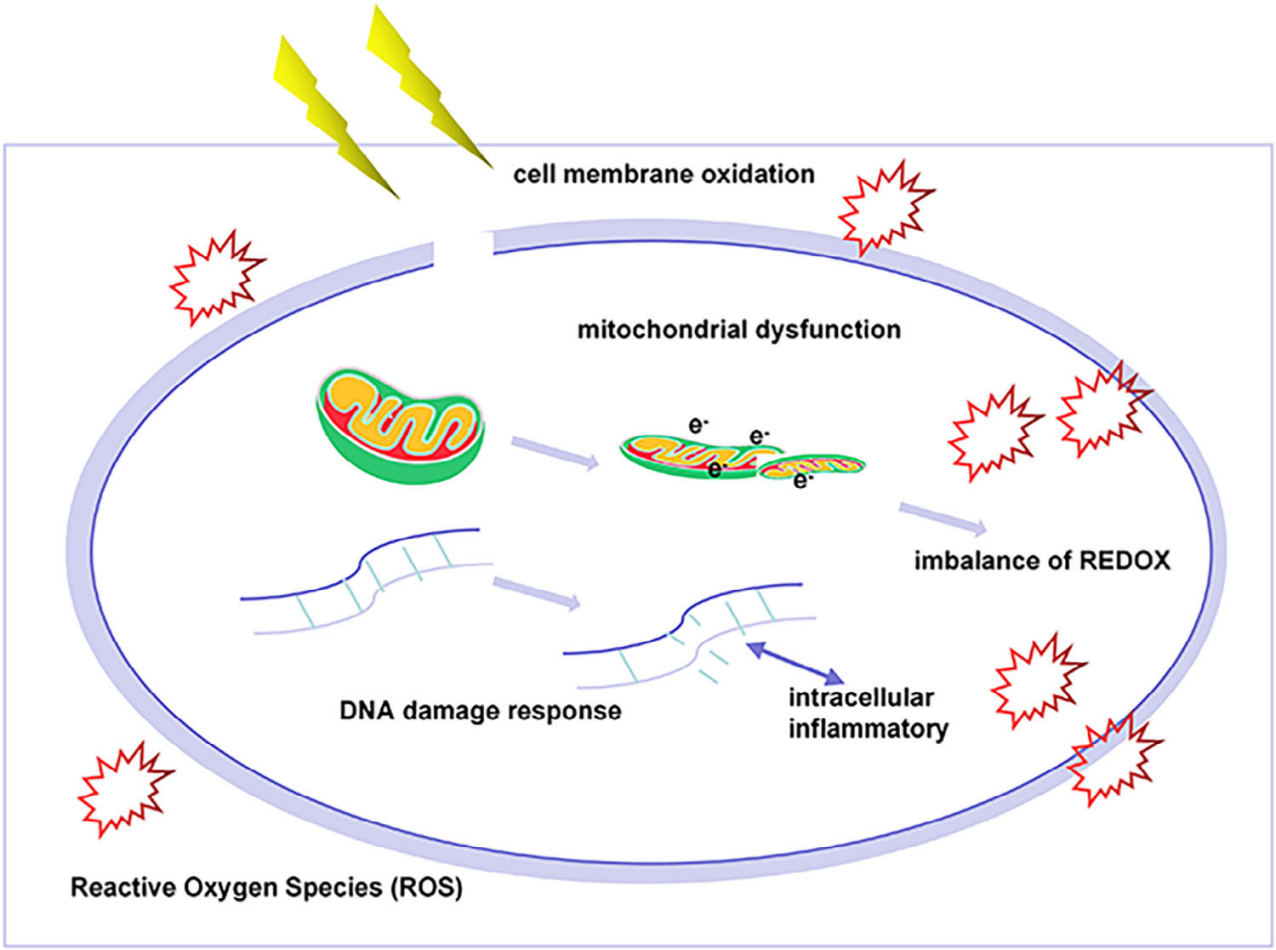
Mechanism of ROS production. IR induces DNA damage, which triggers endogenous inflammatory reactions that generate reactive oxygen species (ROS). Concurrently, this damage leads to mitochondrial DNA destruction, electron leakage (e^−^), and mitochondrial dysfunction, ultimately disrupting the REDOX balance. Additionally, the cell membrane can produce ROS through peroxidation and the disruption of ion permeability.

IR damage significantly elevates both intracellular and extracellular levels of reactive oxygen species (ROS), which triggers cellular oxidative stress responses.^114^ This oxidative stress leads to lipid peroxidation of cell membranes, protein oxidation, and DNA damage, ultimately resulting in cell death and tissue injury.^115^, ^116^ Furthermore, elevated ROS levels during tumor IR therapy can exacerbate inflammatory responses and promote tumor cell proliferation, thereby worsening tissue damage and tumor progression.^117^, ^118^ Consequently, targeted regulation of ROS levels holds substantial potential for preventing and treating RITI. The production of ROS induced by IR involves various biochemical processes, including DNA damage, mitochondrial dysfunction, and cell membrane oxidation^119^, ^120^, ^121^ (Figure 2).

IR can directly or indirectly affect DNA molecules, causing single‐strand break and DSBs as well as base modifications. These DNA injuries activate the DNA damage response (DDR) and DNA repair pathways, which aim to repair the damaged DNA. This process induces the release of cytochrome C and regulates the activation of caspase‐3 and caspase‐9 proteins, triggering a cascade of biological reactions that disturb the cellular REDOX balance.^122^ As a result, ROS generation and release are promoted. Additionally, DNA damage initiates intracellular inflammatory responses and apoptosis, both of which can further contribute to ROS release.^123^ In summary, the mechanisms that promote ROS release following IR‐induced DNA damage involve the regulation of multiple signaling pathways, including the DDR, DNA repair, inflammatory, and apoptosis pathways.

IR significantly impacts the structure and function of mitochondria, leading to mitochondrial dysfunction and increased ROS production.^124^ This process contributes to oxidative stress in tissues. First, alterations in mitochondrial inner membrane permeability disrupt ion balance, which affects mitochondrial membrane potential and promotes ROS generation.^125^ Second, mitochondria serve as cellular energy factories through the mitochondrial respiratory chain. Excessive electron leakage during electron transport is a key contributor to ROS production.^126^ Moreover, mitochondria are crucial sites for intracellular REDOX reactions. Damage to mitochondrial function interrupts these reactions, resulting in increased oxidative stress and further ROS production. Additionally, mitochondrial DNA is a direct target of IR. Damage to mitochondrial DNA can activate intracellular oxidative stress pathways, further elevating ROS levels.^127^ Therefore, maintaining mitochondrial function in the presence of IR is essential for preserving intracellular REDOX balance.

Oxidative stress induced by IR can result in oxidative damage to the cell membrane, thereby enhancing the production of ROS and establishing a positive feedback loop. This process typically involves several mechanisms. First, damage to the cell membrane can cause the opening of ion channels. For instance, the opening of calcium and potassium ion channels allows excess calcium and potassium ions to enter the cell, which subsequently activates enzymes such as NADPH oxidase (NOX) and leads to increased ROS production.^128^, ^129^ Second, membrane damage can trigger the activation of phospholipase. This activation results in the degradation of membrane phospholipids, producing a variety of active oxidized lipids, including peroxides and lipid aldehydes. These compounds further contribute to ROS generation and exacerbate oxidative damage to the cell membrane.^130^ Third, cell membrane injury can disrupt granulosomal function, promoting the production and release of ROS.^131^ Finally, protein oxidation resulting from membrane damage can activate NOX and initiate oxidative stress responses in the endoplasmic reticulum.^132^ This cascade includes ion release, transporter oxidation, activation of the cytochrome P450 system, and mitochondrial dysfunction, all of which further enhance ROS production.

In conclusion, the mechanisms of ROS generation induced by IR are complex and multifaceted. These mechanisms interact with one another, resulting in a substantial increase in intracellular ROS levels. Elevated ROS can cause oxidative damage to critical cellular components, including cell membranes, proteins, and DNA. This damage can lead to various cell fate outcomes, such as apoptosis, disorders in DNA repair, gene mutations, and inflammatory responses. Understanding the mechanisms of ROS production is essential for elucidating cellular responses to IR. Furthermore, this knowledge provides valuable insights for the development of new IR protection strategies that target ROS.

#### 2.2.2 Effects on cellular structures and functions

Upon receiving IR, the levels of ROS in cells increase due to DNA damage, mitochondrial dysfunction, and cell membrane peroxidation. This elevation creates a high oxidative stress environment, exacerbating oxidative damage to proteins, lipids, and nucleic acids. Consequently, normal cellular metabolism and signal transduction are disrupted. Metabolic stress mediated by ROS is implicated in various diseases, including RITI, diabetes, and tumorigenesis.^133^, ^134^, ^135^ Moreover, ROS generated from electron leakage during the electron transport chain can directly impair mitochondrial function. This impairment leads to a decrease in mitochondrial membrane potential and damages the mitochondrial respiratory chain, thereby regulating ATP production and cellular energy metabolism in a feedback loop.^135^, ^136^ When intracellular ROS levels become excessively elevated, the apoptotic pathways are activated, resulting in cell death.^137^ Excessive ROS can also cause structural damage within cells. High levels of ROS lead to protein cross‐linking and oxidative damage to lipids and nucleic acids, which in turn impair organelle function and alter cell structure. An imbalance between ROS production and the activity of the antioxidant system—comprising glutathione, superoxide dismutase, and catalase—further exacerbates oxidative stress. This imbalance ultimately damages cellular structures.^138^ Additionally, ROS can promote the depolymerization of the intracellular cytoskeleton and induce oxidative damage to the cell membrane, adversely affecting cell morphology and motility.^139^

In summary, ROS negatively impact cell structure by damaging biological macromolecules, disrupting the intracellular environment, and promoting apoptosis. To counteract oxidative stress, cells utilize antioxidant enzymes, antioxidants, and various other pathways to clear ROS. Consequently, maintaining the balance of ROS is crucial for cellular homeostasis. This balance is essential for the normal functioning and integrity of cell structure and function.

### 2.3 Inflammatory responses

#### 2.3.1 Cytokine release and immune cell activation

**FIGURE 3:**
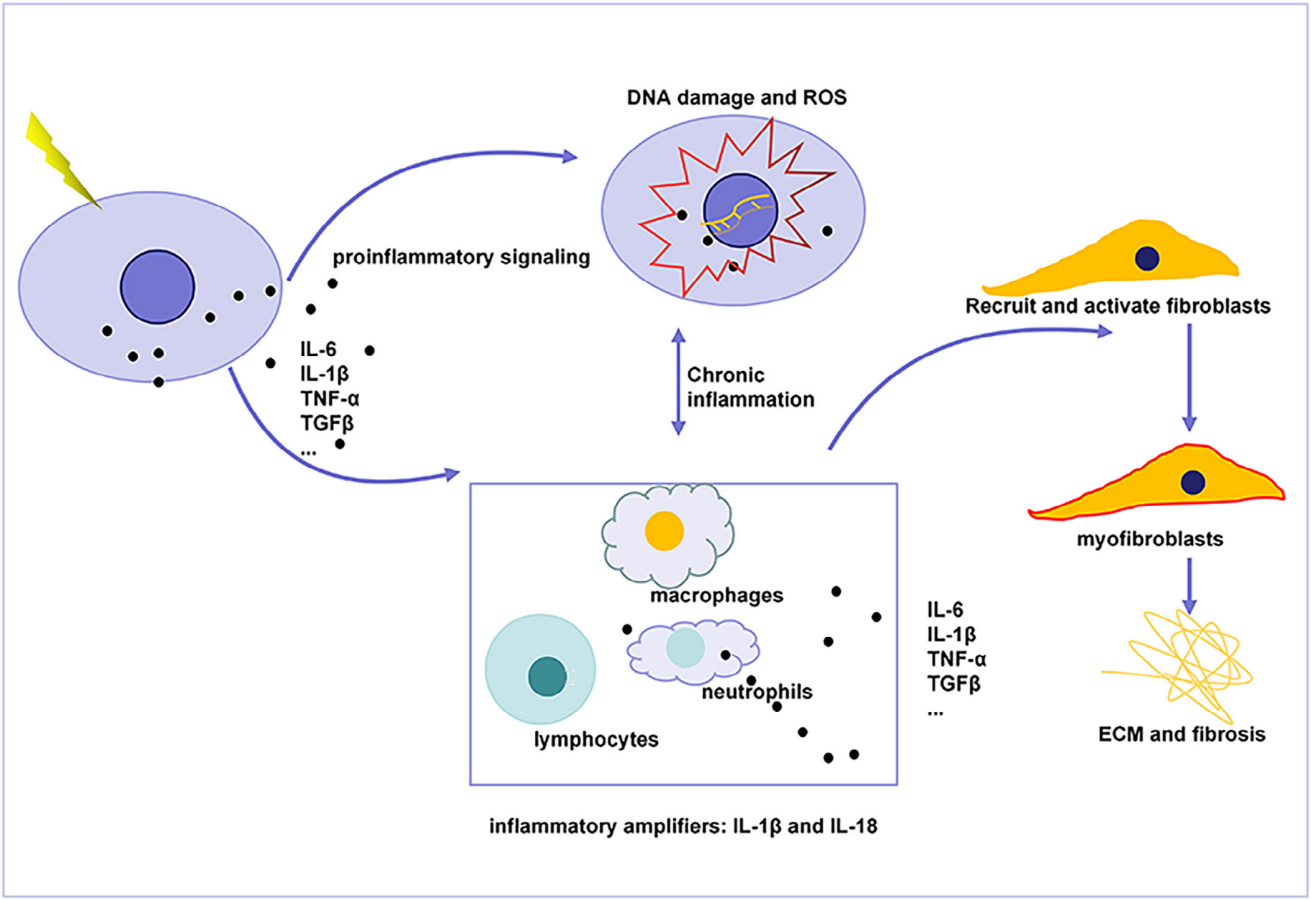
Mechanism of inflammation and fibrosis in RITI. IR can damage target cells, leading to the release of proinflammatory cytokines. This process results in increased DNA damage and elevated levels of reactive oxygen species (ROS). Additionally, it recruits and activates immune cells (macrophages, neutrophils, and lymphocytes), which amplifies inflammation and creates a chronic inflammatory microenvironment. Concurrently, there is an increase in profibrotic signals that recruit and activate fibroblasts, inducing the accumulation of extracellular matrix (ECM) and resulting in fibrosis.

RITI is widely recognized as an inflammatory disease (Figure 3). The injury caused by IR not only damages target cells through mechanisms such as cell senescence, apoptosis, and necrosis but also triggers inflammation in surrounding cells and tissues. RILI is characterized by tissue damage driven by lung epithelial cells. Upon exposure to IR, a significant influx of inflammatory cells occurs at the site of injury. Early in the acute injury phase, neutrophils, eosinophils, lymphocytes, and macrophages aggregate and become activated.^140^, ^141^ Additionally, macrophages play a crucial role in clearing cell debris and necrotic tissue. Exposure of tissue to IR results in DNA damage and triggers a DDR. This response activates various proinflammatory signaling pathways, including NF‐κB, which enhances the tissue's immune response.^142^, ^143^ Target cells affected by IR may mature and release interleukins (ILs) IL‐1β and IL‐18 through the activation of inflammasomes. Both IL‐1β and IL‐18 function as multifunctional immunomodulators and inflammatory amplifiers.^144^ They mediate the initiation of innate and adaptive immune responses, leading to pyroptosis of the affected cells. This process results in the excessive production of proinflammatory cytokines, such as IL‐1α, IL‐6, and tumor necrosis factor (TNF)‐α, which further amplify tissue inflammation.^145^ Additionally, IR can induce cellular senescence.^85^ Senescent cells secrete a range of factors known as the senescence‐associated secretory phenotype (SASP), which includes inflammatory cytokines like IL‐1α, IL‐6, and TNF‐α. The SASP contributes to the diffusion of senescence and establishes paracrine senescence, ultimately leading to chronic inflammation and tissue damage.^146^ During the progression of RITI, inflammatory cells activated by the initial injury propagate the inflammatory response. They secrete cytokines, chemokines, and growth factors that recruit circulating immune cells to the injured site. IL‐1α, IL‐1β, TNF‐α, transforming growth factor‐beta (TGF‐β), and PDGF are commonly observed in models of RITI.^147^

#### 2.3.2 Chronic inflammation and fibrosis

Activated immune cells play a crucial role in wound healing and the fibrosis response by secreting various proinflammatory cytokines and profibrocytokines. In the early stages of RITI, the recruitment of macrophages, neutrophils, and eosinophils promotes the expression of ILs and other proinflammatory cytokines, thereby mediating early inflammation.^148^, ^149^, ^150^ This inflammatory response gradually diminishes over time, which may facilitate wound healing and contribute to fibrosis. The timing of these inflammatory events is critical, as it influences the progression of RITI. Macrophages, in particular, are central to the development of RIPF.^151^ They maintain a chronic inflammatory microenvironment in damaged tissues. Early activation of M1 macrophages releases TGF‐β, chemokines, and other factors that activate fibroblasts, promoting their proliferation, migration, and differentiation into myofibroblasts.^152^, ^153^ These myofibroblasts are resistant to apoptosis, resulting in abnormal deposition of ECM components, such as collagen and matrix metalloproteinases (MMPs). Conversely, M2‐type macrophages stimulate an anti‐inflammatory mechanism that inhibits the release of inflammatory mediators and promotes tissue repair.^152^, ^154^ However, the appearance of M2 macrophages is delayed in the progression of RITI. Once present, they enhance fibrosis by secreting multiple cytokines, further facilitating the transformation of fibroblasts into myofibroblasts and aiding in the healing of tissue injury.

### 2.4 Cell death pathways

#### 2.4.1 Apoptosis

PCD, commonly referred to as apoptosis, is a genetically regulated process that facilitates cell death in response to specific stimuli. This mechanism is crucial for maintaining the stability of the body's internal environment. Apoptosis can be initiated through two primary signaling pathways.^155^ The first is the extrinsic pathway, mediated by death receptors. In this pathway, the binding of extracellular death receptors to their corresponding ligands activates the intracellular apoptosis executor, caspase.^156^ The second pathway is the intrinsic, or mitochondria‐mediated, signaling pathway. This pathway is triggered by factors such as DNA damage, hypoxia, and growth factor deficiency, which regulate mitochondrial membrane permeability and lead to the release of apoptosis activators, ultimately activating caspase.^157^

Apoptosis plays a vital role in various biological processes, including embryonic development, immune regulation, and tumor progression.^158^ Numerous studies have demonstrated that IR induces DNA damage, resulting in the inactivation of the G2/M cell cycle checkpoint. This inactivation, coupled with the activation of ROS and caspase, leads to significant G2/M and S phase cell cycle blocks, culminating in apoptosis.^159^ The expression of the death receptor Fas, which is upregulated by IR, is both time‐ and dose‐dependent, showing a positive correlation with the rate of apoptosis.^160^ p53, a key protein in cell cycle regulation, accumulates and becomes phosphorylated and activated in response to DNA damage caused by IR. This stabilization of p53 promotes DNA repair. However, if the damage is irreparable, p53 can trigger the Golgi apparatus to release stored death receptors, which then bind to death ligands and induce the expression of the proapoptotic gene Bax, further enhancing apoptosis.^161^ Research indicates that alveolar type II epithelial cells undergo apoptosis early in response to IR, effectively removing damaged cells. However, the subsequent loss of these epithelial cells disrupts alveolar structures and leads to other forms of inflammatory cell death. This progression hampers the body's ability to clear damaged cells promptly, resulting in severe inflammatory responses that exacerbate RILI.^162^ In a healthy organism, an increase in myofibroblasts within lung tissue activates the apoptotic regulatory system. Conversely, in patients with RIPF, myofibroblasts exhibit resistance to apoptosis, accompanied by multifactorial ECM remodeling. Notably, studies have shown that treatment with the small molecule dye IR‐780 can inhibit fibroblast differentiation, promote apoptosis, and alleviate RIPF.^163^ Therefore, inducing early apoptosis in damaged epithelial cells and promoting fibroblast apoptosis following IR exposure may represent a novel approach for preventing and treating RILI.

#### 2.4.2 Necrosis

Cell necrosis is an irreversible and non‐programmed form of cell death. It typically occurs when cells are exposed to external factors such as chemicals, IR, trauma, or infection. This process results in the rapid expansion and rupture of cells, leading to the release of cellular contents and the subsequent induction of an inflammatory response. In contrast to apoptosis, which preserves tissue homeostasis and function, necrosis can cause significant tissue damage and dysfunction, contributing to the progression of various diseases.^164^

The characteristics of necrosis include the breakdown of the nucleus and cell membrane, the dissolution of organelles, and the leakage of cellular contents along with proinflammatory molecules.^164^ At the molecular level, the activation of receptor interacting serine/threonine protein kinase 1 (RIPK1) is crucial for the necrotic process.^165^ RIPK1 can be activated by death receptors, such as TNF receptors and Fas.^166^ Once activated, RIPK1 recruits RIPK3, which phosphorylates mixed lineage kinase domain‐like pseudokinase (MLKL). This phosphorylation leads to the formation of MLKL oligomers that translocate to the plasma membrane, resulting in increased membrane permeability and cell destruction.^167^ IR is a direct inducer of cell necrosis. It causes damage to critical cellular components, including DNA, proteins, and lipids, through mechanisms such as breakage, crosslinking, and oxidation. Severe damage from IR triggers either the apoptotic or necrotic pathways, ultimately leading to cell death. The necrosis pathway can be activated in response to IR, influenced by various receptors, including apoptosis receptors (e.g., TNF and Fas receptors) and toll‐like receptors.^168^, ^169^ The cellular response to IR is complex and varies based on factors such as IR dose, type, and the specific characteristics of the affected cells.^170^ Therefore, understanding the mechanisms and effects of cell necrosis is essential in the contexts of IR protection and radiotherapy. This knowledge can help mitigate damage to normal cells during treatment.

#### 2.4.3 Autophagy

Autophagy is a cellular self‐digestion process that decomposes and removes damaged organelles and excess cellular components. This mechanism plays a crucial role in regulating the cell's stress response, thereby maintaining the stability and health of the internal cellular environment. During autophagy, cells utilize enzymes to break down damaged organelles, proteins, and cell membranes. The resulting breakdown products can be reused to facilitate cellular regeneration and repair.^171^ Additionally, autophagy enables cells to cope with various stressors, including hypoxia, starvation, and infection, thus protecting them from damage.^172^ Dysregulation of autophagy has been linked to the development of numerous diseases, such as cancer, neurodegenerative disorders, and metabolic disorders.^173^ Consequently, studying autophagy not only elucidates important regulatory mechanisms within cells but also offers new perspectives for disease treatment and prevention.

The mechanism of autophagy induced by IR involves multiple signaling pathways and molecular processes.^174^, ^175^ IR initiates and executes autophagy by inducing DNA damage and oxidative stress. This process activates protein kinase signaling pathways and regulates the expression of autophagy‐related genes. Specifically, IR can cause single‐strand break and DSB in DNA, as well as base damage, leading to the production of ROS and subsequent oxidative stress. These damage and stress signals activate autophagy pathways within cells. Furthermore, IR activates various protein kinases, including AMPK, mTOR, and PI3K, which promote the initiation and execution of autophagy.^176^ Additionally, IR influences the expression of several autophagy‐related genes, such as Beclin‐1 and LC3. Changes in the expression of these genes directly regulate the progression of autophagy.^177^ During RITI, enhanced autophagy aids in the clearance of damaged proteins and organelles, thereby protecting cells from further harm. Moreover, autophagy regulates apoptosis, the cell cycle, and DNA repair processes, influencing the repair and regeneration of tissues affected by IR. Investigating these mechanisms deepens our understanding of the effects of IR on cells and provides novel insights for treating related diseases. Therefore, studying the role and regulatory mechanisms of autophagy is of great significance in the prevention and treatment of RITI.

## 3 RITI AND Sc‐seq

### 3.1 A brief of RITI

**FIGURE 4:**
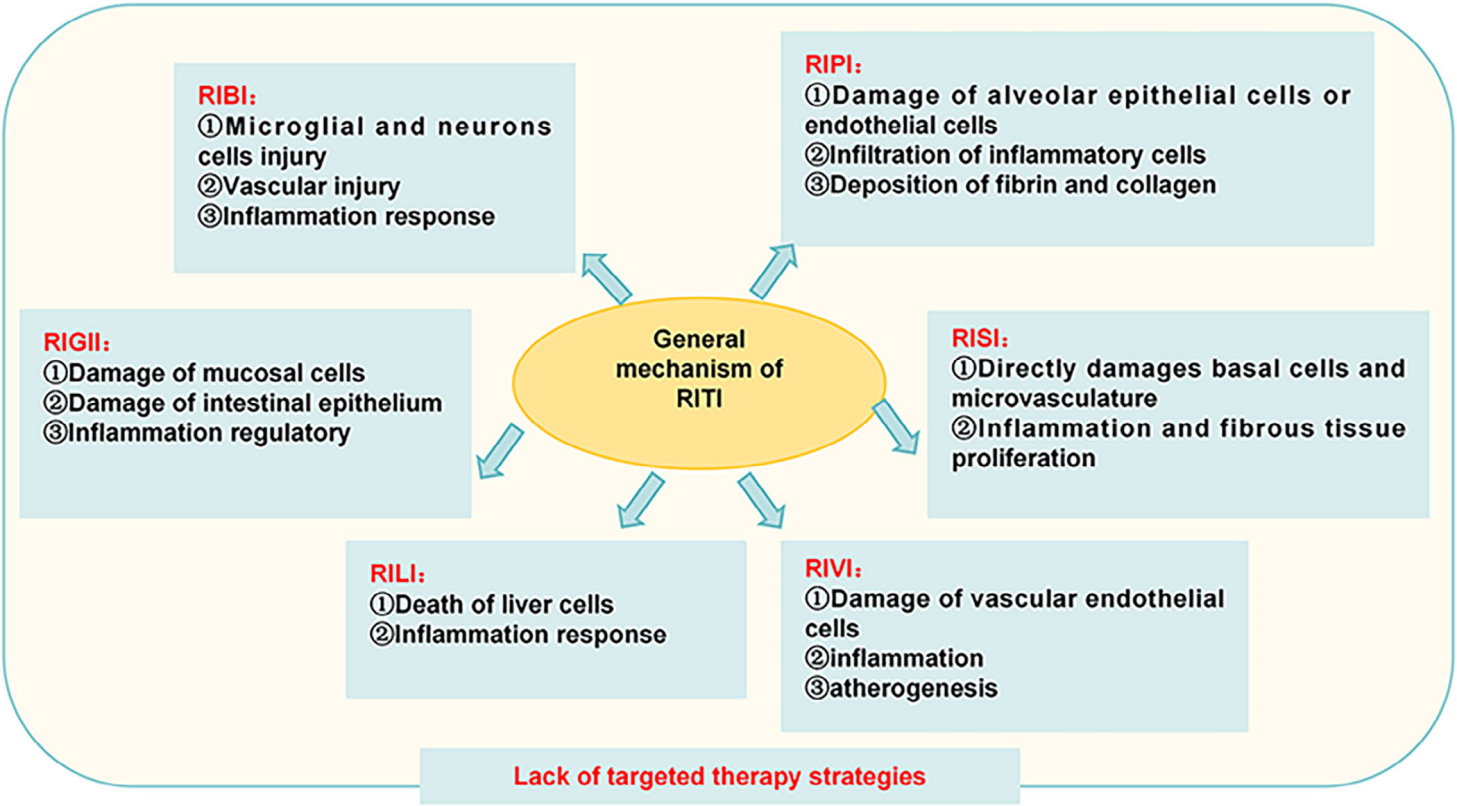
The general mechanism of radiation‐induced tissue injury (RITI). The complexity of signaling pathways in the occurrence of RITI. The main pathological process of RITI: target cell death induced by directly DNA damage or ROS, the inflammation response or fiber production. RIBI, radiation‐induced brain injury; RIGII, radiation‐induced gastrointestinal injury; RILI, radiation‐induced liver injury; RIPI, radiation‐induced pulmonary injury; RISI, radiation‐induced skin injury; RIVI, radiation‐induced vascular injury.

Radiotherapy is an important cancer treatment that aims to kill tumor cells through IR, either to cure the disease or to alleviate symptoms. However, while IR is effective in targeting tumor cells, it also inevitably damages surrounding normal tissues, so it greatly limits administered dose of radiotherapy.^22^, ^178^, ^179^, ^180^, ^181^, ^182^ To standardize the assessment of RITI (Table 1), the National Cancer Institute, the Oncology Radiation Therapy Consortium, and the European Center for Cancer Research collaborated in developing the RITI grading system. This system categorizes more than 4000 kinds of adverse reactions in 28 organs based on subjective manifestations, objective signs, treatment measures, and detection methods. RITI can be divided into acute injury and chronic injury depending on the timing of occurrence, such as early IR‐induced pneumonia (RIP) can be relieved by hormone and late IR‐induced pulmonary fibrosis (RIPF).^183^ Dehydration treatment and reducing cranial pressure are effective in treating acute and early brain damage.^184^ Advanced brain injury, characterized by IR‐induced brain necrosis, is the most common clinical manifestation after IR exposure.^185^ Consequently, targeting the early stage of RITI as a promising strategy to alleviate these following symptoms. The response of different tissues to IR varies significantly, resulting in RITI presenting with both common mechanisms and tissue‐specific symptoms and pathological features. Mechanistically, IR directly cause DNA damage^186^, ^187^ and indirectly release ROS to damage cells.^11^, ^188^ And in the occurrence of RITI process usually accompanied by DDR^187^, ^189^ and tissue damage repair response, which reshapes the tissue microenvironment.^190^, ^191^ However, once the repair is excessive, scar or fibrosis will occur, which will aggravate the tissue damage. RIPF is characterized by excessive activation of lung tissue repair, primarily caused by abnormal myofibroblast activation leading to increased ECM deposition.^192^, ^193^, ^194^ Striking a balance between damage and repair in RITI is crucial for developing effective therapeutic strategies. Thus, it is pertinent to review the pathological character and detail mechanism of the well‐known and extensively studied RITI, as the understanding of the mechanism is one of the basis of determining therapy strategy (Figure 4).

**TABLE 1:** The typical clinical symptoms and external causes of radiation‐induced tissue injury.

Classification	Clinical symptoms	External causes
Radiation‐induced brain injury	Progressive dementia	Radiotherapy for head and neck malignancies
	Cognitive dysfunction	
Radiation‐induced gastrointestinal injury	Nutrient absorption disorders	Radiotherapy for abdominal malignancies
	Impairment of gastrointestinal barrier function	
Radiation‐induced liver injury	Upper gastrointestinal bleeding	Radiotherapy for upper abdominal and thoracic tumors, preoperative radiotherapy of bone marrow transplantation
	Liver failure	
Radiation‐induced pulmonary injury	Radiation‐induced pneumonia	Thoracic malignant tumor radiotherapy, preoperative radiotherapy of bone marrow transplantation
	Radiation‐induced pulmonary fibrosis	
Radiation‐induced skin injury	Skin infection	All the tumor radiotherapy
	Necrosis and fibrosis	
Radiation‐induced vascular injury	Atherogenesis	All the tumor radiotherapy
	Severe ischemia	
	Organ failure	
Radiation‐induced cardiac injury	Pericardial effusion	Radiotherapy for thoracic malignancies
	Pericarditis and myocarditis	
Radiation‐induced hematopoietic system injury	Hematopoietic disorder	All the tumor radiotherapy
	Hematopoietic failure	
Radiation‐induced bladder and ureteral injury	Circulating inflammation	Clinical pelvic malignant tumor radiotherapy
	Bladder injury	

### 3.2 A brief of Sc‐seq

Sc‐seq have revolutionized the field of cellular biology by enabling detailed analysis of cellular heterogeneity at the single‐cell level in both cells and tissues. These technologies aim to unveil specific cell subtypes or unique cellular metabolic signatures through the uniquely expressed gene sets or metabolic groups, thereby providing sequencing sample‐specific genetic information along with spatial context. The main technologies utilized in Sc‐seq include single‐cell RNA sequencing (scRNA‐seq) for gene expression analysis,^49^ single‐cell assay for transposase‐accessible chromatin with high‐throughput sequencing for chromatin structure analysis,^195^, ^196^ Single‐cell proteomics sequencing for proteome analysis,^197^, ^198^ single‐cell metabolomic sequencing for metabolome analysis, and spatial transcriptome analysis which has seen rapid advancements in recent years.^199^ By its nature, Sc‐seq allows for the identification and screening of healthy cell subsets in diseased tissues, as well as for the determination of whether the basic physiological functions of the entire cell population are normal. Additionally, it can pinpoint the presence or absence of cells expressing specific genes that are crucial for supporting therapeutic applications. Notably, Sc‐seq technology has played a pivotal role in breakthroughs related to tumor mechanisms, tumor immunotherapy, tissue inflammation, as well as embryonic development and evolution (Table 2). Moreover, the rapid advancement of Sc‐seq assays has facilitated the discovery of disease biomarkers and the development of targeted drugs, further enhancing precision medicine approaches.

**TABLE 2:** Classification and application of single‐cell sequencing.

Method	Basic principle	Application	Anticipated goal	References
Single‐cell RNA sequencing	Gene expression	Cancer research Stem cell differentiation neurobiology Developmental biology Immune regulation Biomarker Therapy targets	Tracing disease specific cell subpopulations Analyze the mechanism of disease occurrence and development Unique cellular metabolic signatures Explore the combination drug target	^48^, ^49^
Single‐cell assay for transposase‐accessible chromatin with high‐throughput sequencing	Chromatin structure			^195^, ^196^
Single‐cell proteomics sequencing	Proteome			^196^, ^197^, ^198^
Single‐cell metabolomic sequencing	Metabolome	Biomarkers Therapy targets		^199^

### 3.3 IR‐induced brain injury

IR‐induced brain injury (RIBI) is a common complication of radiotherapy for head and neck malignancies.^200^, ^201^, ^202^ About of 50% patients undergoing this treatment will experience varying degrees of progressive dementia and cognitive dysfunction.^9^, ^203^, ^204^ The main mechanisms of RIBI include direct injury to neurons and glial cells, vascular injury, and inflammatory response.^205^, ^206^, ^207^

#### 3.3.1 Mechanisms and cellular responses

Microglial, innate immune cells in the brain, play a crucial role in the pathogenesis and progression of central nervous system damage and diseases.^208^ When brain tissue is exposed to IR, it induces alterations in the somatosome, protrusions, shape, and phagocytic capacity of microglia (MG), resulting in their abnormal activation. Studies have confirmed that approximately 90% of glial cells in the brain are morphologically abnormal and accompanied by shortening or disappearance of protrusions when brain tissue is irradiated with a single 8 Gy IR.^209^ It was found that cranial IR activates the STAT3 signaling pathway, induces astrocyte sustained activation, reactive hyperplasia, and hypertrophy, and increases expression of intracellular vascular endothelial growth factor (VEGF).^210^ After IR, MG are abnormally activated, triggering the secretion of an array of proinflammatory cytokines such as monocyte chemoattractant protein‐1 (MCP‐1), TNF‐α, ILs (IL‐1β, IL‐18 and IL‐1а), and granulocyte‐macrophage colony‐stimulating factor (GM‐CSF), and dampening the release of anti‐inflammatory cytokines such as CD86, CD32, CD206, and IL‐10, thereby amplifying the neuroinflammatory response. Furthermore, studies have observed that microglial cell numbers transiently increase post‐IR, followed by a decrease, stabilizing around the 5th day after IR.^211^, ^212^ Depletion of MG can reduce the incidence of brain injury induced by helium IR. IR could induce the apoptosis of neurons to directly decrease the number of neurons. After IR, the expression of miR‐711 is upregulated, and a variety of survival and DNA repair mechanisms are negatively regulated, neuronal apoptosis and senescence mechanisms are activated, thus inhibiting DNA repair and promoting neuronal degeneration.^213^ However, in IR‐combined injury, the loss of nerve cells is serious.^214^ Celecoxib can effectively inhibit the apoptosis of cerebral vascular endothelial cells.^215^ When MG are damaged, IR‐induced neuronal damage can be exacerbated, which directly leads to impaired neuronal function, and on the other hand, this cell‐to‐cell interaction can induce an inflammatory cascade response in the central nervous system, thereby exacerbating the neuroinflammation associated with RIBI.

The pathogenesis of RIBI including vascular injury and microcirculation disorders, resulting in cerebral ischemia, aggravated capillary permeability, increased effusion of tissue fluid, and ultimately cerebral infarction and cerebral edema. Multiple signaling pathways likely contribute to vascular injury and microcirculation disorders in the progression of RIBI. Orai3‐mediated activation of SOCE plays an important role in IR‐induced injury to rat brain microvascular endothelial cells and brain injury, and knocking out SOCE can alleviate vascular injury.^216^ Besides, the highly expression of VEGF is a key reason of RIBI, and many studies have identified that Bevacizumab alleviates brain edema symptoms caused by IR brain necrosis through inhibiting VEGF and acting on vascular tissue around the brain necrosis area.^217^

Exposing with IR promotes an inflammation response in RIBI, releasing cytokines such as TNF‐α, IL‐1β, and IL‐6.^211^, ^218^ In rhesus monkey models of graded whole‐brain IR, RIBI was primarily attributed to neuroinflammation, an inflammatory response that was primarily due to activation of MG.^219^ Microglial activation promoted by high expression of MCP‐1 can further recruit circulating monocytes into the central nervous system and exacerbate neuroinflammation.^220^ After exposing with IR, the p65 subunit of the NF‐κB complex underwent nuclear translocation and significantly upregulated NEMO and significantly decreased NF‐κB regulation‐α.^221^, ^222^ Pregabalin inhibits IR‐induced NF‐κB activation and microglial inflammatory response by inhibiting the HMGB1‐TLR2/TLR4/RAGE signaling pathway.^223^ Besides, targeting GBP5/NF‐κB/NLRP3 Signal Axis by a synthetic steroid alleviates MG activation and proinflammation cytokines release in RIBI.^224^ In wild rodents, miR‐124 in extracellular vesicles derived from human neural stem cells can eliminate IR‐induced cognitive dysfunction and neuroinflammation, alleviating RIBI.^225^ These mechanism studies in the regulation of inflammation provide new directions for targeting RIBI.

#### 3.3.2 Insights from Sc‐seq

Vascular endothelial senescence plays an important role in RIBI.^226^ Whole‐brain IR‐induced endothelial cell senescence has been shown to cause chronic damage to the blood–brain barrier (BBB) and a reduction in microvascular density through scRNA‐seq analysis. Interestingly, the drug ABT263s has demonstrated the ability to effectively reverse the BBB injury resulting from whole‐brain IR in mice.^227^ Furthermore, studies have confirmed a series of changes in MG post‐IR exposure. Specifically, study has shown that steady‐state genes in MG in the hippocampus are significantly downregulated and inflammation‐related genes are upregulated after IR, thereby promoting the phagocytic function of MG. Notably, despite exhibiting early impacts on MG, the alterations induced by IR return to baseline levels within 1 week.^228^ In addition, Tang et al.^229^ used scRNA‐seq and scTCR sequencing techniques to found the CCL2/CCL8 chemokines produced by MG. This chemokine mediates CCR2‐positive or CCR5‐positive and CD8‐positive T cell infiltration of brain injury tissue in RIBI mice, emphasizing the key role of MG in mediating adaptive immune dysregulation. The cumulative evidence suggests potential therapeutic strategies for addressing RIBI.

### 3.4 IR‐induced gastrointestinal injury

IR‐induced gastrointestinal injury (RIGII) is one of the common complications of radiotherapy for abdominal malignancies.^230^, ^231^ IR above 4 Gy can cause gastrointestinal syndrome.^232^ The main mechanism is that IR destroys the small intestinal crypts, promotes the damage of mucosal cells, destroys the integrity of the intestinal epithelium, finally leading to nutrient absorption disorders in the intestine, impairing gastrointestinal barrier function.

#### 3.4.1 Mechanisms and cellular responses

Various factors contribute to the pathophysiology of RIGII. The damage of mucosal is a general symptom of RIGII often culminating in lethal sepsis and gastrointestinal bleeding.^233^, ^234^ Relevant studies have confirmed the crucial involvement of exosomes, apoptosis, and ferroptosis in RIGII.^235^ Ginseng extract ginsenoside Rk1^236^ has been found to exert an antiapoptotic role by inhibiting the PI3K/Akt/mTOR pathway. Additionally, baicalin^237^ can also antagonize mitochondrial apoptosis of intestinal cells. Furthermore, IR exposure triggers the production of ACSL4 by intestinal flora, and lead to intestinal cell ferroptosis. However, troglitazone has shown promising results in antagonizing this process.^238^

Intestinal stem cells are essential for the maintenance of intestinal epithelial cell function, and IR can induce intestinal stem cell injury.^233^, ^239^, ^240^ Studies have shown that the use of HIF upregulating factor FG‐4592^241^ and transplantation of Lgr5‐positive rectal stem cells,^242^, ^243^ as well as activation of vitamin D receptors in the intestine,^244^ can restore intestinal stem cells, promote intestinal epithelial cell regeneration, and protect against RIGII. Studies on melatonin have also shown that melatonin 5 mg/kg can effectively prevent RIGII.^245^ Vanillin derivative VND3207 can protect intestinal epithelial cells from IR‐induced injury by activating DNA–PKcs both in vivo and in vitro.^246^ Moreover, microtubule‐associated serine/threonine kinase 1,^247^ acidic sphingomyelin enzyme and ceramides secreted by endothelial cells,^248^ hepatocyte growth factor, TNF stimulating gene 6 released by mesenchymal stem cells,^249^ as well as interferon regulatory factor 1^250^ can all protect the intestines from RIGII.

Apart from these mechanisms, disturbance of intestinal flora and inflammation also plays an important role. It has been confirmed that the activation of NLRP3 inflammasome and infiltration of neutrophils contribute to the occurrence and development of injury. To combat inflammation and oxidative stress in RIGII, interventions such as sitagliptin,^251^ rosiglitazone,^252^ arabinoxylan rice bran,^253^ antibiotic pretreatment,^254^ dasatinib,^255^ CXCR2 inhibitors,^256^ and so on, have been proven to be efficacy in providing protection and alleviating damage. These findings underscore the complex interplay of molecular pathways and cellular processes involved in RIGII pathogenesis.

#### 3.4.2 Insights from Sc‐seq

After exposure to IR, changes occur in immune cell subpopulations, leading to imbalances in the immune ecosystem. Specifically, IR‐specific immune cells are widely involved in intercellular communication, with CD8‐positive T cells serving as a central component. CD8‐positive T cells are closely related to cell ferroptosis, and the interaction between vascular endothelial cells and immune cells is also a pivotal point in RIGII.^257^, ^258^ To gain a comprehensive understanding of the cellular dynamics at different stages of RIGII, researchers use scRNA‐seq to construct a detail panoramic dynamic cellular map.^259^ Analysis of the scRNA‐seq data revealed an increase in resident macrophages secreting anti‐inflammatory factor IL‐10, as well as an increase in inflammatory macrophages secreting proinflammatory factors TNFα, IL‐1, and IL‐6 after IR. Moreover, the activation of the Arl6ip1 gene has been shown to regulate the differential sensitivity of intestinal epithelial cells to IR. Knockdown of Arl6ip1 leads to increased DNA injury induced by IR in intestinal epithelial cells, inhibiting their proliferation. Bmi1‐negative and GFP‐positive cells and Prox1‐positive cells, part of the intestinal endocrine lineage, have been demonstrated to possess intestinal stem cell activity. These cells are crucial in maintaining homeostasis and facilitating regeneration following endothelial cell injury. Specifically, they serve as injury‐induced intestinal stem cells that bolster the renewal of intestinal epithelial cells.^260^ Setd4‐positive reserve stem cells have the capacity to endure IR injury, promoting the restoration of the intestinal epithelium and the reestablishment of crypts.^261^ In addition, researchers have established a proliferative intestinal organoid containing eight components. This organoid contains a population of regenerative stem cells, with a focus on Lgr5‐positive stem cells, and Clu‐positive stem cells. These stem cells have molecular characteristics that mimic intestinal epithelial regeneration and are capable of generating proliferative crypts following injury, hence providing protection against intestinal injury.^262^ Through studies utilizing Sc‐seq, we have gained a more thorough insight into the pathogenesis of RIGII and have been able to identify the key target cells and potential targets for therapeutic intervention of RIGII with greater precision.

### 3.5 IR‐induced liver injury

IR‐induced liver injury (RILI) is one of the serious complications caused by radiotherapy for upper abdominal and thoracic tumors, as well as preoperative radiotherapy of bone marrow transplantation. In severe cases, RILI may result in upper gastrointestinal bleeding and liver failure.^263^ The primary pathophysiological mechanism involves IR‐induced hepatic vein occlusion, liver cell death, inflammation response, extensive fibrin hyperplasia, and ultimately the development of cirrhosis and hepatic failure.

#### 3.5.1 Mechanisms and cellular responses

IR has the capability to directly cause DSBs and damage other cell structures, ultimately leading to the death of liver cells. Moreover, IR can stimulate cells to generate a significant amount of ROS, which can subsequently harm the cell membrane and mitochondria, resulting in various forms of cell death such as apoptosis, necrosis, or ferroptosis.^264^, ^265^ Another route through which RILI may contribute to liver cell death is by triggering inflammatory and immune responses.^265^, ^266^ The tissue damage resulting from IR exposure can activate inflammatory cells and immune cells, prompting the release of inflammatory mediators and cytokines that spark an inflammatory and immune reaction within liver tissue. This, in turn, exacerbates liver cell death and tissue damage. For instance, the JAK/STAT/NF‐E2‐related factor 2 (NrF2)‐mediated inflammatory response can exacerbate ferroptosis in liver cells.^265^ Thus, gaining a more profound comprehension of these mechanisms holds promise for the development of enhanced treatments and preventative strategies.

In the progression of RILI, mechanisms involving inflammation response play a crucial role alongside other pathways. Studies have pointed to the significance of the STING signaling pathway in the progression of RILI due to IR‐induced DNA damage and the subsequent cGAS–STING pathway activation.^267^ Furthermore, the ALKBH5–HMGB1–STING axis has also been implicated in the occurrence and development of RILI.^268^ The METTL3–TEAD1–STING axis has been identified as another mechanism contributing to RILI by facilitating pyroptosis.^266^ Additionally, the involvement of the NF‐κB pathway in RILI has been demonstrated, with the ALKBH5–TIRAP–NF‐κB signaling pathway showing substantial impact through TIRAP demethylation and downstream NF‐κB activation in inducing RILI.^269^ Notably, curcumin^270^ has been proven to effectively antagonize NF‐κB. In addition, TNF is closely related to RILI,^271^ and TNF‐α can serve as a biomarker^272^ of the RILI detection. These findings illustrate the various cellular and molecular mechanisms underlying the inflammatory response induced by IR in RILI.

#### 3.5.2 Insights from Sc‐seq

Although there have been many studies trying to analyze the etiology of RILI, due to the complex mechanism of RILI and the current lack of therapeutic targets, it is of critical need for further research into the pathogenesis of RILI and finding targets for prevention and treatment. Song et al.^273^ utilized scRNA‐seq technology to identify the pivotal role of interferon I in the process of acetaminophen‐induced acute liver injury. Through their investigation, they also unveiled the presence of Trem2‐positive macrophages capable of triggering the intracellular IFN‐I synthesis pathway. This activation process fosters the production of CSF‐1 by senescent neutrophils, eventually leading to the maturation of Trem2‐positive macrophages and the development of RILI. Thus, these findings underscore the significance of interferon I in the pathogenesis of RILI, shedding light on potential targets for prevention and treatment.

### 3.6 IR‐induced pulmonary injury

Studies have shown that about 15% of thoracic malignant tumor radiotherapy patients will have different degrees of IR‐induced pulmonary injury (RIPI). This injury is mainly affected by the patient's age, IR volume and dose, whether there are underlying diseases, and whether they are combined with other risk factors. The pathological changes of the injury can be roughly divided into three stages,^274^ the first stage is the early period, also known as the incubation period, the second stage is the acute exudation period, also known as the pneumonia period, and the third stage is the late period, also known as the fibrosis period. The clinical manifestations were early RIP and late RIPF. The main mechanism is the damage of alveolar epithelial cells or endothelial cells, infiltration of inflammatory cells, and deposition of fibrin and collagen.

#### 3.6.1 Mechanisms and cellular responses

When exposed to IR, the lung tissue undergoes RIPI as a result of complex interactions among multiple cell types and factors. Alveolar epithelial cells and endothelial cells emerge as pivotal target cells contributing to the development of RIPI. The impairment of these target cells, manifested in various forms, can result in the reduction of lung epithelial stem cells, disruption of alveolar structure, and initiation of tissue inflammation, ultimately leading to the progression of RIPI. The epithelial/endothelial‐to‐mesenchymal transition (EMT) of alveolar epithelial cells and endothelial cells plays an important role in this process.^275^ IR can induce overexpression of METTL3, increase the m6A modification level of FOXO1 mRNA, and recruit YTHDF2 to degrade and downregulate FOXO1, thereby activating AKT and ERK pathways, and ultimately promoting the progression of EMT.^276^ Hsp27 also activates the IkBα–NF‐κB pathway, resulting in increased expression of Twist, IL‐1β, and IL‐6^277^. The activation of myeloid zinc finger 1 (MZF1) can inhibit the expression of miR‐541‐5p and also participate in the EMT process.^278^ The C‐Raf inhibitor GW5074 mitigated the EMT process in mice by inhibiting the C‐Raf/Twist1 signaling pathway.^279^ RIPI involves various mechanisms, with apoptosis,^280^ senescence,^16^, ^281^ and pyroptosis^282^ playing crucial roles. Targeting lung vascular endothelial cell apoptosis with basic fibroblast growth factor can help alleviate RIPI,^280^ and inhibition of Rac1^283^ can effectively inhibit apoptosis and production of ROS. Senescence of alveolar epithelial cells is a significant factor in the onset and progression of RIPF. Effective improvements in lung function and alleviation of RIPF symptoms can be achieved by eliminating senescent alveolar epithelial cells and administering antisenescent drugs.^284^ The involvement of STING‐dependent self‐DNA‐driven pyroptosis in RIPI has been confirmed. Furthermore, inhibiting the NLRP3 inflammasome pathway using ACT001 has shown to ameliorate RIPI,^285^ and inhibit NLRP3 inflammasome pathway by ACT001 has shown to ameliorate RIPI.^286^ Activation of the P62–Keap1–NRF2 pathway was found to mitigate cell ferroptosis in RIPI.^287^, ^288^, ^289^ Prevent the loss of epithelial cells after irradiation or replace important mediators related to immune and fibroblast activity produced by epithelial cells, and enhance mesenchymal differentiation of alveolar stem cells, both mediate the occurrence of RIP.^290^, ^291^

Tissue inflammation plays an important role in this process. Studies have shown that the NLRP3 inflammasome plays a crucial role.^286^, ^292^ It has been confirmed that the dsDNA–cGAS–STING–NLRP3 axis is involved in the occurrence of RIP and RIPF, and eliminating cGAS or STING, and inhibiting or depleting NLRP3 inflammasome can alleviate the degree of inflammation and fibrosis in mouse pulmonary tissue. Using eNAMPT monoclonal antibodies to interfere with the eNAMPT/TLR4 inflammatory pathway also effectively mitigates the severity of RIP.^293^, ^294^ Other studies have shown that lymphocytes^295^, ^296^ and neutrophils^297^ are associated with the risk of RIP. The decreased levels of peripheral blood lymphocytes and CD4‐positive T lymphocytes were closely related to the increased risk of RIP, and the greater the degree of CD4‐positive T lymphocytes decreased, the greater the risk of RIP. The interaction between macrophages and epithelial cells is also very important in RIPF. IR can induce senescence of macrophages,^298^ and senescent macrophages produce SASP^299^ and promote the occurrence of RIP. TGF‐β1‐mediated inflammatory response affects the progression of RIPI. Studies have shown that drugs such as pirfenidone^300^ can reduce the production of TGF‐β1, effectively alleviate pathological features and biochemical markers, reduce the expression of inflammatory cytokines, inhibit the progression of RIP, and mitigate RIPI.^301^ Monophosphoryl lipid A (MPLA), herbal extract PM014, statin analog DRDE‐30, and nicaraven^302^ can stimulate the production of exosomes, downregulate NF‐κB and TGF‐β1, and effectively alleviate RIPI.^303^

In the progression of RIPF, the abnormal deposition of ECM including TGFβ, MMPs (MMP2, MMP9, etc.), fibrin, and collagen plays a crucial role. Exposure of lung tissue to IR triggers the abnormal multiplication and activation of fibroblasts, leading to increased numbers and activity of myofibroblasts. Subsequently, this induces the aberrant deposition of ECM components such as fibrin and collagen, thereby promoting the progression of RIPF.^304^ In addition, lung epithelial cells and endothelial cells contribute to the proliferation of fibroblasts through EMT,^26^, ^305^ and lung epithelial cells can release a large number of inflammatory factors to activate fibroblasts.^306^ Additionally, macrophages and other immune cells recruit circulating fibroblasts to the lung by releasing TGFβ and other profibrotropic factors, further amplifying the fibrotic phenotype in the lung tissue.^307^

#### 3.6.2 Insights from Sc‐seq

Until now, there is no specific therapy method to RIPI, which forces application of Sc‐seq to RIPI essential. In 2023, research has found that mutations at positions 53 and 54 in the TAD2 region of P53 protein transcription activation promoted the directed differentiation of transitional cells to AT1 by acting on transitional cells, facilitating tissue repair and antitumor development by utilizing scRNA‐seq and sc‐ATAC sequencing.^308^ Furthermore, the activation of Sox9‐positive cells has been linked to promoting lung tissue regeneration through the activation of the PI3K/AKT pathway, as revealed by scRNA‐seq analysis.^309^ In addition, some scholars have found that there is obvious heterogeneity among lung tissue cells after IR, especially the internal heterogeneity of immune cells.^310^ Endothelial cells have been found to express high levels of adhesion molecules like CXCL16, which aid in recruiting proinflammatory Th cells to sites of inflammation, exacerbating lung injury. The activation and upregulation of the cGAS–STING pathway, inflammatory factors CCL5, ICAM1, PF4, TNF‐α, and so on have been confirmed to be significantly implicated in RIPF, and oxidative stress is also involved in the occurrence and development of RIP.^311^, ^312^ Studies have highlighted that the expression of multiple mitochondrial genes is increased after IR. By employing Sc‐seq technology to investigate lung tissue injury induced by programmed death 1 or radiotherapy in cancer treatment, researchers have been able to establish a comprehensive lung injury atlas, shedding light on the cytodynamics of different cell components and treatment‐related transcriptome alterations.^313^ In addition, FLASH IR can reduce the induction of inflammatory genes, mitigate the level of DNA damage in normal cells, and protect lung progenitor cells from death caused by excessive IR, effectively reducing IR‐induced mortality.^314^ Based on these single‐cell results, key intervention targets and cell groups within crucial signaling pathways of RIPI have been more accurately identified.

### 3.7 IR‐induced skin injury

IR‐induced skin injury (RISI) mainly occurs within 2 weeks after epidermal cells are radiated. The sebaceous glands, hair follicles, and basal cells in the skin are highly sensitive.^315^ Severe RISI may have repeated skin infection and necrosis, which greatly affects the quality of life of patients. The main mechanism is that IR directly damages basal cells and microvasculature, induces the tissue inflammation and promotes a large amount of fibrous tissue proliferation.

#### 3.7.1 Mechanisms and cellular responses

Qiu et al.^316^ conducted a study utilizing whole genome sequencing to identify that the zinc transporter ZIP9, regulated by DNA methylation, is expressed via TGF‐β signal pathways, thereby promoting IR‐induced skin fibrosis (RISF). In addition, the activation of nuclear receptor coactivating factor 3 (NCOA3) following IR exposure contributes to the regulation of skin IR sensitivity, cell cycle progression, and the prevention of DDR, ultimately leading to the development of skin melanoma.^317^ Noteworthy mechanisms related to oxidative stress, inflammatory response, and cell apoptosis also significantly contribute to RISI that cannot be underestimated. For instance, studies have found that NLRP3 inflammasomes play a protective role in defending against RISF by attenuating cGAS–STING signaling pathways.^318^ Nrf2 has been shown to ameliorate RISF by activating antioxidant enzymes.^319^ Moreover, the upregulation of interferon alpha‐inducible gene 6 has been demonstrated to promote cell proliferation, reduce apoptosis, and diminish the production of ROS after IR, weakening RISI.^320^ Stem cell involvement and microbial metabolism are also crucial in these processes. A type of adipose‐derived stem cells has been shown to exhibit antiapoptotic effects and alleviate IR dermatitis by inhibiting the expression of tissue protease F (CTSF).^321^ In addition, Huang et al.^322^ highlighted the pivotal role of the microbial metabolite axis in RISI. IR alters the distribution and function of microorganisms on the skin surface, thereby affecting the metabolic processes of the three major nutrients and inducing skin injury. Additionally, post‐IR exposure, fatty acid synthase regulation by miR‐206‐3p and miR‐378a‐3p is implicated in the pathogenesis of skin injury.^323^ Notably, FLASH radiotherapy has been observed to reduce severe skin toxicity compared with conventional radiotherapy.^324^ Moreover, the activation of the autophagy gene Atg7 has shown promise in attenuating RISI.^325^

Excessive production of ROS occurs during IR, leading to damage to biomacromolecules, oxidative stress, and inflammation.^326^ In this context, dressings containing EGT–NaHA have demonstrated an ability to scavenge free radicals effectively, thereby reducing oxidative stress, apoptosis, and inflammation both in vitro and in vivo.^326^ Importantly, IR exposure has been found to decrease the level of laminin B1 in epidermal keratinocytes. Furthermore, senescent cells, in addition to entering a stable growth arrest state, exhibit a SASP characterized by the release of various cytokines, chemokines, growth factors, and proteases, which contribute to tissue inflammation.^327^ Notably, skin exposed to IR becomes more susceptible to microbial infections, particularly those caused by Staphylococcus aureus.^328^ Neutrophils play a crucial role in the immune response by capturing and destroying invading microorganisms through phagocytosis, which can lead to an inflammatory response. Moreover, chronic skin reactions following IR exposure often involve fibrosis of the cutis and subcutis.^329^, ^330^ Moreover, chronic skin reactions following IR exposure often involve fibrosis of the cutis and subcutis. The molecular mechanisms driving skin fibrosis are complex and involve the excessive production of collagen and ECM components by dermal fibroblasts.^330^ These processes contribute to the structural changes observed in the skin following IR exposure.

#### 3.7.2 Insights from Sc‐seq

Paldor et al.^331^ utilized scRNA‐seq to examine the critical factors driving RISI. After exposure to IR, senescence‐related IL‐6, IL‐1, and IL‐17 signaling pathways were found to be upregulated, while the expression of CCR6 was identified as key in mediating immune cell migration. Inhibition of senescence and immune migration can improve RISI effectively. Using scRNA‐seq technology, Zhang et al.^332^ investigated early RISI and found that fatty acid metabolism significantly increased in the early RISI, especially unsaturated fatty acids. Downregulation of ACADVL was shown to heighten the radiosensitivity of keratinocytes and skin fibroblasts, whereas silencing of ACADVL led to increased expression of proteins associated with apoptosis and pyroptosis after IR exposure. These findings propose a novel strategy for targeted prevention and therapeutic intervention in managing RISI.

### 3.8 IR‐induced vascular injury

IR‐induced vascular injury (RIVI) is the most common complication of radiotherapy and the basis of other RITI.^333^ Vascular endothelial cells are extremely sensitive to IR. The main mechanism of RIVI is that IR directly damages vascular endothelial cells, inflammation and atherogenesis.

#### 3.8.1 Mechanisms and cellular responses

During radiotherapy, one of the most crucial disorders that emerges is endothelial vascular injury. This injury directly damages vascular endothelial cells, induces their apoptosis, and leads to hyaloid degeneration of small arteries.^334^ It has been suggested that the involvement of vascular smooth muscle cells (VSMCs) senescence plays a role in the pathogenesis of IR‐induced atherosclerosis. Insightfully, IR induces VSMCs senescence by regulating the NF‐κB/CTCF/p16 pathway.^335^ Subsequently, the deposition of many lipids in the subcutaneous injury promotes atherogenesis in the large and medium arteries, causing significant obstruction of microcirculation. This obstruction further reduces the density of blood vessels, eventually leading to severe ischemia of local organs. As a result, a vicious circle is formed, ultimately culminating in organ failure. To defend against vascular damage, it is crucial to rely on strong antioxidants.^336^

#### 3.8.2 Insights from Sc‐seq

In recent years, with the development of scRNA‐seq technology, the mechanism of RIVI has been the subject of further investigation. By using scRNA‐seq technology, Benadjaoud et al.^337^ found that RIVI was closely related to endothelial cell senescence. They revealed the presence of significant heterogeneity in the senescent status of endothelial cells in human umbilical veins after 7 days of IR exposure. Specifically, all endothelial cells highly expressed the senescence gene CDKN1A. The decrease of von Willebrand factor and EMT process are correlated with the high senescence state of endothelial cells. Relevant studies^338^ have highlighted the role of IR‐induced senescence in CCR6‐positive Th17 cells in promoting the secretion of IL‐8 and VEGF‐A, which can induce injury to normal tissue, potentially contributing to tumor recurrence and metastasis. Inflammation also plays a crucial role in RIVI, with IL‐18 identified as a key player in RITI through the IL‐18/IFN‐γ/ROS inflammatory pathway.^339^ Consequently, therapies targeting senescence and inflammation have the potential to effectively mitigate RIVI and alleviate IR‐induced organ injury.

### 3.9 Others

#### 3.9.1 Mechanism of IR‐induced cardiac injury

IR‐induced cardiac injury (RICI) is one of the serious complications of radiotherapy for thoracic malignancies, with the pericardium being particularly susceptible to IR exposure. The predominant clinical presentations include pericardial effusion, pericarditis, and myocarditis.^340^ The primary mechanism involves various pathological processes, such as the increase in pericardial collagen fibers, myocardial fibrosis, endothelial cell damage in coronary vessels, generation of ROS, and oxidative accumulation of low‐density lipoprotein due to IR. These factors collectively promote accelerated atherosclerosis, disturbance of cardiac microcirculation, serious insufficiency of myocardial blood perfusion, and eventually myocardial cell ischemia, hypoxia, degeneration, and necrosis. It has been found that Atp5f1c lysine 55 acetylation (Atp5f1c K55‐Ac) can lead to post‐IR cardiometabolic dysfunction, thereby mediating IR‐induced cardiomyocyte senescence.^341^ Ferroptosis also plays an important role in RICI.^342^ Notably, studies have confirmed that l‐histidine and its secondary metabolites imidazole propionate^343^ and fullerenols from intestinal microorganisms can significantly mitigate RICI.^344^

#### 3.9.2 Mechanism of IR‐induced hematopoietic system injury

IR can directly injure the bone marrow, spleen, and other important hematopoietic organs, leading to the blood cell production disorders, and ultimately death. The sensitivity of the hematopoietic system to IR has prompted research on the mechanisms involving hematopoietic stem cells (HSCs). It has been shown that the deletion of RPRM can upregulate the expression levels of p‐EGFR, p‐DNA–PKcs, and p‐STAT3 after IR, significantly reduce the damage of the IR‐induced hematopoietic system injury (RIHSI), and also preserve the hematopoietic regeneration potential of mouse HSCs to alleviate the IR‐induced DNA injury.^345^ Additionally, γ‐ray exposure activates voltage‐dependent anion channels (VDAC), increases mitochondrial DNA release into the cytoplasm, activates the cGAS signaling pathway, and leads to hematopoietic inhibition.^346^ The protective effect on HSCs can be achieved by using VDAC1 inhibitors such as DIDS and cGAS synthetase inhibitors. Recombinant human thrombopoietin has also been shown to affect the ROS/p53/p21/p16 pathway after IR to regulate hematopoietic remodeling and HSC circulation disorder, improve IR‐induced myelosuppression in mice, and mitigate HSCs injury.^346^ The receptor‐interacting protein kinase 3 (Ripk3) signaling pathway inhibits the development of IR‐induced leukemia in mice by regulating HSCs during stress.^347^ Moreover, studies have indicated that myeloid‐directed progenitors derived from human peripheral blood mononuclear cells can induce host HSC repair and regeneration through exosomal paracrine signaling, promoting bone marrow recovery by enhancing self‐renewal and proliferation. This mechanism serves to alleviate hematopoietic acute response syndrome following IR exposure.^348^

#### 3.9.3 Mechanism of IR‐induced bladder and ureteral injury

IR bladder and ureteral injury is also one of the common complications of clinical pelvic malignant tumor radiotherapy. Groves et al.^349^ found that the RAS inhibitor captopril could effectively prevent changes in urination patterns from chronic radiotherapy improve the proportion deterioration of neutrophils and monocytes mediated by circulating inflammation and alleviate radiotherapy‐induced advanced bladder injury.

## 4 PROSPECT OF Sc‐seq IN RITI

**FIGURE 5:**
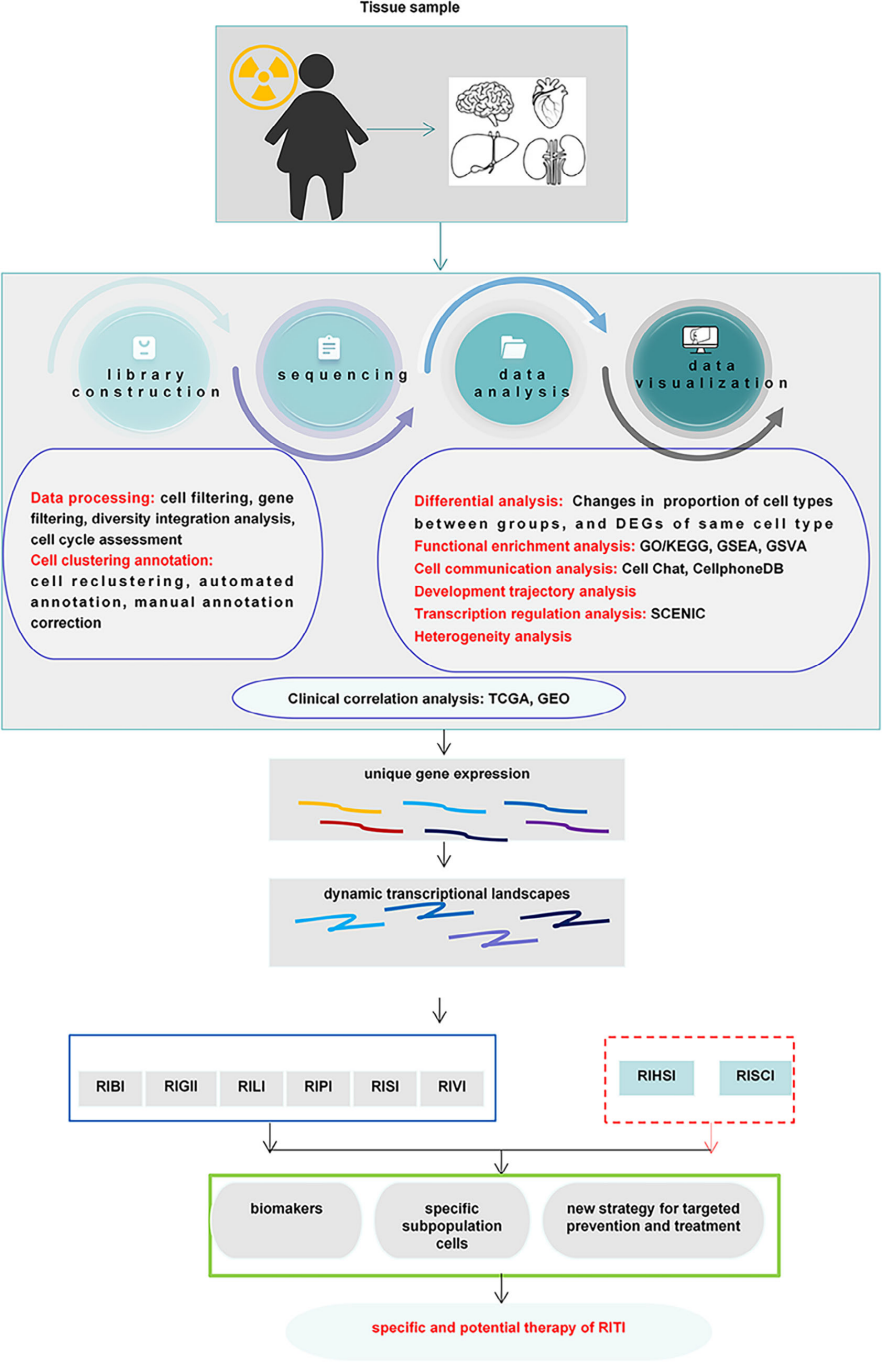
The role and progress of single‐cell sequencing (Sc‐seq) in identifying potential novel biomarkers, specific subpopulation cells and new strategy for targeted prevention and treatment of radiation‐induced tissue injury.

RITI research has traditionally focused on elucidating the fundamental mechanisms underlying this condition, with limited specific therapeutic targets were identified (Table 3). This gap in knowledge has posed a significant challenge for radiotherapy. To address this issue, a comprehensive exploration of the pathogenesis of RITI from a more detailed and precise perspective is crucial for the development of targeted therapeutic interventions. One promising technology that has emerged as a powerful tool for advancing our understanding of RITI pathogenesis and potential treatment options is Sc‐seq. By offering a high‐resolution platform for characterizing RITI at the single‐cell level, Sc‐seq holds the potential to elucidate the molecular pathways driving disease progression and reveal novel cellular subpopulations. While the application of Sc‐seq in RITI research has made valuable contributions, there are still numerous promising avenues for further investigation in this field (Figure 5).

**TABLE 3:** Therapeutic strategies and inhibitors for radiation‐induced tissue injury.

Type	Drug	Category	Mechanism	Application	References
RIBI	Celecoxib	NSAIDs	Inhibition of cerebral vascular endothelial apoptosis	Clinical application	^215^
	Pregabalin	Calcium ion channel regulators	Inhibition of NF‐κB and microglial inflammation	Clinical application	^223^
	miR‐124	Micromolecule	Eliminating radiation‐induced neuroinflammation	animal experiments	^350^
	ABT263s	Bcl‐2 protein family inhibitors	Antisenescence	animal experiments	^227^
RIGII	Rk1	Ginseng extract	Inhibition of PI3K/Akt/mTOR pathway	animal experiments	^236^
	Baicalin	Flavonoids	Resistance to mitochondrial apoptosis	Clinical application	^237^
	Troglitazone	PPAR gamma agonists	Resisting ferroptosis by inhibiting ACSL4	Clinical application	^238^
	Sitagliptin	DPP‐4 inhibitor	Anti‐inflammatory and antioxidant	Clinical application	^251^
	Rosiglitazone	Thiazolidinone diketone insulin sensitizers	Anti‐inflammatory and antioxidant	Clinical application	^351^
	MGN‐3	Arabinoxylan rice bran	Anti‐inflammatory and antioxidant	Clinical trial	^253^
	Cocktail (Abx)	Antibiotic	Anti‐inflammatory and antioxidant	Clinical trial	^254^
	Dasatinib	Antineoplastic drug	Anti‐inflammatory and antioxidant	Clinical application	^255^
	SB225002	CXCR2 inhibitor	Anti‐inflammatory and antioxidant	Animal experiments	^256^
	FG‐4592	HIF upregulation factor	Activation of vitamin D receptors in the intestine	Clinical application	^241^
	VND3207	Vanillin derivatives	Activate DNA–PKcs	Animal experiments	^246^
RILI	Curcumin	Acidic polyphenols	Antagonism against NF‐κB	Clinical application	^270^
RIPI	ALT‐100	eNAMPT mAb	Interference with eNAMPT/TLR4 pathway	Clinical trial	^293^
	GW5074	Selective C‐Raf inhibitors	Inhibiting the C‐Raf/Twist1 signaling pathway	Clinical trial	^279^
	Pirfenidone	Pyridone compounds	Reduce TGF‐β1	Clinical application	^300^
	MPLA	Agonists for Toll like receptor 4	Stimulate the production of exosomes	Clinical trial	^303^
	PM014	Herbal extracts	Regulating NF‐κB and TGF‐β1/NOX4 pathways	Animal experiments	^288^
	DRDE‐30	Statin analogues	Inhibition of TGF‐β/Smad pathway	Animal experiments	^289^
	Nicaraven	Hydroxyl radical scavenger	Inhibition of NF‐κB and TGF‐β/Smad pathways	Clinical trial	^302^
	NSC23766	Rac1 inhibitor	Inhibiting apoptosis and ROS production	Animal experiments	^283^
RIBUI	Captopril	ACEI	Preventing changes in the ratio of neutrophils to monocytes	Clinical application	^348^
RIHSI	DIDS	VDAC1 inhibitor	Inhibition of mitochondrial DNA release	Animal experiments	^346^
	rHuTPO	Biological agents	Affect the ROS/p53/p21/p16 pathway	Clinical application	^352^

Abbreviations: NSAIDs, nonsteroidal anti‐inflammatory drugs; Rk1, Ginseng extract ginsenoside Rk1; rHuTPO, recombinant human thrombopoietin; eNAMPT, extracellular nicotinamide phosphoribosyltransferase; MGN‐3, MGN‐3 arabinoxylan.

First, general idea of RITI mechanism is to identify injury cells, which contributes to the treatment of RITI. Future studies employing Sc‐seq should strive to fully elucidate the cellular diversity and unique cell subpopulations and identify their distinct gene expression profiles, which may make great contribution for targeted therapeutic strategies of RITI. Second, applying Sc‐seq to specific therapy of RITI in radiotherapy is an area of great significance. Elucidating RITI at a single‐cell resolution may yield invaluable insights into their unique biology and reveal potential vulnerabilities for targeted therapies in tumor radiotherapy progress. However, there are challenges that need to be addressed to fully leverage the potential of Sc‐seq in RITI research. Issues such as the high cost of the technology and the complexity of data analysis present significant barriers for researchers seeking to implement this approach. Overcoming these obstacles through cost optimization and improved analytical strategies is essential to maximizing the impact of Sc‐seq in advancing our understanding of RITI and developing effective therapeutic interventions.

## 5 CONCLUSION AND PROSPECTS

RITI not only affects the effectiveness of radiotherapy but in severe cases can even pose a threat to the patient's life. Therefore, it is significant to study the IR‐induced mechanisms of injury related to different organizations. Furthermore, the critical roles of cell injury mechanisms such as senescence, apoptosis, and pyroptosis‐related proteins in RITI have been well established. Additionally, it has been recognized that inflammation, immune cell migration, and fibrogenesis play key roles in RITI, thereby providing a foundation for the identification of potential therapeutic targets.

**FIGURE 6:**
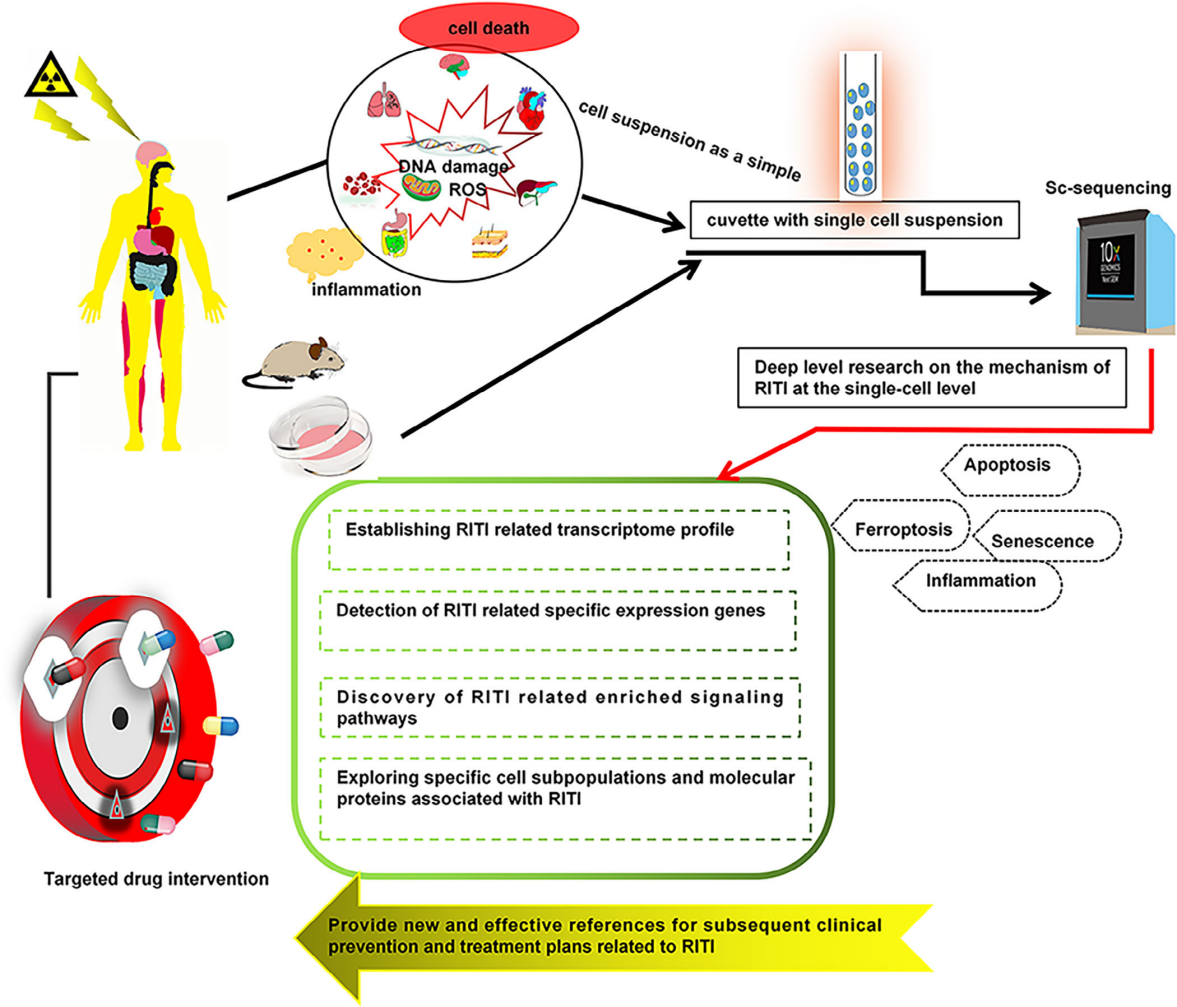
The conclusion of this review. Overall, Sc‐seq has transformed research on radiation‐induced tissue injury (RITI) by enabling comprehensive analysis of clinical manifestations, pathological features, molecular mechanisms, and regulatory networks at the single‐cell level. This approach has facilitated a deeper understanding of the targeted intervention pathways and therapeutic drugs for RITI. The insights gained from Sc‐seq have opened new avenues for developing personalized therapies that have the potential to significantly improve patient outcomes.

To delve into these mechanisms, Sc‐seq, in conjunction with fundamental experiments, has been employed to monitor cellular changes following IR exposure. Through this approach, a deeper understanding of the diverse cell subpopulations’ responses has been achieved, leading to the creation of comprehensive dynamic single‐cell maps representing the various stages of IR‐induced injury. The utilization of Sc‐seq technology has enabled the identification of specific factors and cells with therapeutic potential, thereby enhancing our knowledge base on RITI^353^ (Figure 6).

Moving forward, the continued advancement of Sc‐seq technology holds promise for expanding research horizons, facilitating breakthroughs, and offering robust technical support for clinical and basic investigations into RITI. As Sc‐seq emerges as a cornerstone in the scientific research domain, its widespread adoption is inevitable. Efforts aimed at improving the accessibility and cost‐effectiveness of this technology are vital to realizing its full potential in the context of RITI treatment strategies. With ongoing progress in science and technology, the evolution of Sc‐seq technology is poised to unlock new avenues for targeted interventions in clinical RITI, paving the way for transformative developments in the field.

## AUTHOR CONTRIBUTIONS

Lin Zhou and Jiaojiao Zhu were the major contributors in writing the manuscript. Yuhao Liu collected the literature. Ping‐Kun Zhou and Yongqing Gu revised the manuscript. All authors read and approved the final manuscript.

## CONFLICT OF INTEREST STATEMENT

The authors declare that they have no conflict of interest.

## ETHICS STATEMENT

Not applicable.

## Data Availability

Not applicable.
